# Pomegranate Dieback in Italy: New Insights into the Etiology of the Disease

**DOI:** 10.3390/jof12020125

**Published:** 2026-02-10

**Authors:** Silvio Tundo, Donato Gerin, Angela Bolzonello, Rocco Caracciolo, Luca Sella, Francesco Faretra, Francesco Favaron, Stefania Pollastro

**Affiliations:** 1 Dipartimento di Territorio e Sistemi Agro-Forestali (TESAF), Università degli Studi di Padova, Viale dell’Università 16, 35020 Legnaro, Italy; angela.bolzonello@unipd.it (A.B.); caracciolo.rocco@gmail.com (R.C.); luca.sella@unipd.it (L.S.); francesco.favaron@unipd.it (F.F.); 2 Dipartimento di Agronomia, Animali, Alimenti, Risorse Naturali e Ambiente (DAFNAE), Università degli Studi di Padova, Viale dell’Università 16, 35020 Legnaro, Italy; 3 Dipartimento di Scienze del Suolo della Pianta e degli Alimenti—Università degli Studi di Bari Aldo Moro, via Amendola 126/A, 70126 Bari, Italy; francesco.faretra@uniba.it (F.F.); stefania.pollastro@uniba.it (S.P.)

**Keywords:** biocontrol agents, dieback, field survey, molecular identification, pathogenicity, phylogenetic analysis, wood canker

## Abstract

Pomegranate dieback is a disease whose etiology remains only partially understood. In this study, surveys were carried out in orchards located in the Apulia, Basilicata, and Veneto regions from 2016 to 2020 with the objective to identify pathogens involved in pomegranate dieback. Six fungal species were isolated from symptomatic trees and identified through morphological and molecular analyses. In addition to the known pomegranate pathogens *Neofusicoccum parvum*, *Diaporthe eres* and *D. foeniculina*, new fungal species, including *Neopestalotiopsis rosae*, *Sporothrix stenoceras*, and one belonging to the *Xenoacremonium* genus, were identified. This study represents the first report of their association with pomegranate plants exhibiting dieback symptoms. When artificially inoculated on pomegranate trees, these fungi caused wood browning, proving their pathogenicity. All fungal species exhibited optimal growth in the temperature range 25–30 °C, although *D. eres* and *N. roseae* showed a good adaptability in the range 5–10 °C. Since some of the identified pathogens were isolated from the same trees, cross-pairing assays were conducted, revealing that these fungi can coexist within the same ecological niche while maintaining their viability. Given the need for sustainable management options against these co-occurring pathogens, biological control strategies were evaluated. In vitro experiments demonstrated that both *Bacillus* and *Trichoderma* biological control agents (BCAs) inhibit the investigated pomegranate pathogens, highlighting their potential inclusion in integrated management strategies targeting these newly identified fungal pathogens.

## 1. Introduction

Pomegranate (*Punica granatum* L.) is one of the earliest domesticated fruit crops, with origins tracing back to at least 4000 years ago in central Asia [1]. The global expansion of pomegranate cultivation, including in Italy, is driven by the increasing demand for functional foods and the release of varieties with improved organoleptic characteristics as well as by its adaptability to diverse environmental conditions [2,3,4]. The adaptability is particularly relevant in Italy, where northern, central, and southern regions are characterized by markedly different climatic conditions, ranging from temperate to Mediterranean environments. Together with the expansion of cultivated areas, deterioration in tree health and productivity, often termed “pomegranate dieback”, has been observed. Pomegranate dieback is caused by various fungi and oomycetes [5,6,7,8,9]. The condition, which can lead to plant death, is associated with a range of symptoms including crown, stem, shoot, and branch cankers, stunted growth, diebacks, leaf chlorosis and necrosis, and fruit and root rots. *Coniella granati* (Sacc.) Petr. & Syd is reported as one of the main causal agents of pomegranate dieback worldwide [5,10], and its spread has recently been documented in different Italian regions [9,11,12,13]. In northeastern Italy, pomegranate dieback outbreaks have been linked to the concurrent presence of *C. granati* and *Phytophthora palmivora* [12]. Other pathogens such as *Botryosphaeria dothidea*, *Neofusicoccum parvum*, *Phytophthora pseudocryptogea*, and *Phytopythium vexans* have also been isolated from diseased pomegranate trees, albeit less frequently [12]. The contribution of *B. dothidea* and *N. parvum* in pomegranate wood symptoms has been established in several reports worldwide [14,15,16,17,18]. *Neofusicoccum parvum*, a botryosphaeriaceous fungus reported as opportunistic endophyte [19], is known for causing cankers, diebacks, and fruit rots in various woody plants [20]. Interestingly, *C. granati* has been experimentally demonstrated to exacerbate its pathogenic effect on pomegranate in co-infections with *P. palmivora*, suggesting a synergistic interaction between the two pathogens in pomegranate dieback [21]. Other fungal pathogens, such as *Ceratocystis fimbriata*, *Cytospora punicae* and *Lasiodiplodia theobromae*, have been detected in Asian pomegranate orchards, as causes of wilt, wood cankers and diebacks [22,23,24,25,26] revealing a high diversity of wood-infecting fungal pathogens involved in pomegranate dieback.

Disease control requires careful orchard management practices, including early detection and the adoption of cultural measures that minimize inoculum levels, limit its spread, and prevent stress-inducing events [27]. In addition, to align pomegranate cultivation with environmental and food safety standards [28], the use of synthetic fungicides should be minimized. In Western Europe, protection protocols do not include bare wood treatments, and protection is provided only against leaf and fruit diseases, primarily using natural extracts and biological control agents (BCAs) such as bacteria of the *Bacillus* genus and fungi of the genus *Trichoderma* as alternatives to synthetic fungicides [29,30]. Recently, new non-chemical plant protection products have been registered for use in pomegranate orchards. These include Problad (Certis Belchim, Utrecht, The Netherlands), based on an extract from the cotyledons of lupine plantlets, and the *Bacillus*-based biofungicides such as AMYLO-X^®^ (Biogard, CBC Europe Srl, Grassobbio, Italy), SERENADE^®^ ASO (Bayer SAS, Marle sur Serre, France) and Taegro^®^ (Syngenta Italia, Milan, Italy), authorized for the control of *Botrytis cinerea* and/or *Alternaria alternata*. These trends highlight the need for new protection tools useful for the management of pomegranate dieback.

Given the complex and poorly understood etiology of pomegranate dieback, the objectives of this study were the following: (i) isolate, identify and characterize additional potential pathogens involved in pomegranate dieback in Italy; (ii) assess potential interactions between the identified fungal pathogens; (iii) test the in vitro efficacy of commercial BCAs against these pathogens. With these purposes, pathogen isolations were carried out during field surveys conducted at four different locations. The isolates were identified through morphological and molecular analyses, and their pathogenicity was confirmed on pomegranate trees. The biological features of each fungal species, including colony growth under different temperatures, and evaluation of possible synergistic or antagonistic interactions were assessed in agarized media. Finally, we evaluated the in vitro efficacy of several BCAs in inhibiting these pathogens, assessing their potential for future in vivo studies.

## 2. Materials and Methods

### 2.1. Field Surveys, Sampling and Fungal Isolation

Pomegranate trees showing dieback symptoms were monitored in orchards located in different Italian regions: Apulia (one site in the countryside of Grottaglie, Taranto; 40°30′27.7′′ N 17°24′46.7′′ E), Basilicata (one site in the countryside of Metaponto, Matera; 40°25′49.1′′ N 16°43′49.8′′ E) and Veneto (one site in the countryside of San Biagio di Callalta, Treviso; 45°41′16.8′′ N 12°24′56.6′′ E, and another site in the countryside of Legnaro, Padova; 45°22′07.9′′ N 11°57′22.2′′ E). In Apulia and Basilicata, orchards were planted with five-year-old ‘Wonderful’ pomegranate trees, whereas in Veneto the orchards were planted with three-to-five-year-old ‘Wonderful’, Acco’, and ‘Parfianka’ pomegranate trees. Disease prevalence was assessed through visual inspection of the orchards. For each orchard, three plots of 15–20 pomegranate trees arranged along the right diagonal of the field were examined, and the percentage of trees with dieback symptoms (Prevalence, P; %) was calculated.

For each orchard, three representative pomegranate trees showing the presence of wood cankers and dieback symptoms at intermediate levels of severity were selected for sampling. Branches, stems, crowns and roots were visually inspected. Shoots and branches were examined for blights, cankers, and dieback symptoms; crowns were inspected for cankers and rots; and roots were assessed for necrotized tissues and rots. These tree organs were also transversely sectioned, and only those characterized by the presence of internal wood browning were transferred to the Department of Soil, Plant and Food Sciences (DiSSPA, University of Bari Aldo Moro, Bari; Apulia and Basilicata orchards) and to the Department of Land, Environment, Agriculture and Forestry (TESAF, University of Padova, Legnaro; Veneto orchards) for fungal isolation. Wood samples were surface sterilized by washing them in 70% ethanol for 1 min, followed by 1% sodium hypochlorite solution for 3 min, and rinsing them in sterile distilled water three times. Small pieces of tissue (approximately 5 × 5 mm) were cut from the margin of symptomatic wood and plated onto Potato Dextrose Agar PDA, either as a commercial preparation (BD-Difco, Sparks, MD, USA, pH 5.6) or prepared in house as follows: infusion from 200 g peeled and sliced potatoes kept at 60 °C for 1 h, supplemented with 2% dextrose and 2% European bacteriological agar (LLG labware, Meckenheim, Germany) per liter, and adjusted at pH 6.5. The plates were incubated at 25 °C in the dark for three days. Emerging fungal colonies were subcultured by transferring hyphal tips onto fresh PDA or malt extract agar [(MEA; 2% malt extract (Oxoid Thermofisher, Rodano, Italy) and 2% European bacteriological agar (LLG labware)] to obtain pure isolates for subsequent identification.

### 2.2. Morphological and Molecular Identification

Colony morphology (e.g., pigmentation, size, shape, texture) and the production of their reproductive structures (acervuli, perithecia, pycnidia) were observed by visual examination and under a stereomicroscope (MZ125, Leica, Solms, Germany). In addition, spores (asexual and sexual spores) were observed under an optical microscope (Laborlux 12, Leitz, Ostfildern, Germany) equipped by a micrometer. For each isolate, the size of at least 100 spores was measured.

For all isolates, the growth rate was evaluated at 25 °C in the dark on PDA, MEA, Oatmeal agar (OA, BD-Difco, Sparks, MD, USA) and water agar [WA, 2% European bacteriological agar (LLG labware)], to compare fungal growth under nutrient-rich and nutrient-limited conditions. For *Diaporthe eres* and *D. foeniculina*, WA supplemented with autoclaved fennel stems, and for *Neopestalotiopsis rosae*, WA supplemented with pine needles, were also used to enhance asexual sporulation. Mycelial plugs of 7 mm in diameter were collected from the edges of actively growing colonies and used to inoculate three replicated Petri dishes containing the above-reported media. The orthogonal diameters of developing colonies were measured at 2 and 4 days after inoculation (DAI) for all isolates, and the daily growth rate was calculated.

DNA was extracted from mycelium scraped from three-day-old cultures using the NucleoSpin Plant II (Macherey-Nagel GmbH & Co.KG, Düren, Germany). The ITS1-5.8S-ITS2 rDNA internal transcribed spacer (ITS) region, translation elongation factor 1-α (*TEF1*), calmodulin (*cmdA*), β-tubulin (*TUB2*), and large subunit (LSU) of rDNA were amplified for one or all isolates with the primers listed in Table S1. PCR amplifications were performed in 15 μL reaction volume containing 1× buffer (Colorless Go-Taq Flexi, Promega, Milan, Italy), 2.5 mM MgCl_2_ (Promega), 100 nM of each dNTP (Promega), 100 nM of each primer, 1 U of Taq polymerase (Promega), and 100 ng of DNA template. The PCR program consisted of an initial denaturation (94 °C, 3 min), followed by 35 cycles of denaturation (94 °C, 1 min), annealing (55 °C or 58 °C, 30 s; Table S1) and extension (72 °C, 1 min), with a final extension step (72 °C, 10 min). The PCR products were separated and visualized on a 1.5% agarose gel and custom sequenced by external service (Macrogen, Seoul, Republic of Korea; BRM Genomics, Padova, Italy). Sequences were analyzed using BLASTn (version 2.17.0) against both the NCBI GenBank nucleotide database (https://www.ncbi.nlm.nih.gov, last access: 15 December 2025) and the sequences derived from type material.

The Maximum likelihood (ML)-phylogenetic analysis was performed as described in [31], using the combined sequences of ITS-*TEF1*-*TUB2* for *D. eres* and *D. foeniculina*, as well as for *N. rosae*, ITS-LSU-*TUB2* for *Sporothrix stenoceras* and ITS-LSU-*TEF1* for *Xenoacremonium* sp. (Figure S1). MEGA11 was used to infer the best model of nucleotide substitution for the dataset via the Tamura-Nei model and the nearest neighbor interchange heuristic search method. The branch stability was determined by 1000 bootstrap replicates.

### 2.3. Pathogenicity Assays

The first pathogenicity test was conducted at TESAF Department with the fungal species not previously reported on pomegranate in Veneto region, namely *D. foeniculina* (TESAF_DF1), *N. rosae* (TESAF_NR1), *S. stenoceras* (TESAF_SS1), and *Xenoacremonium* (TESAF_X1). The second pathogenicity assay was conducted at the DiSSPA Department with all the fungal species under study. In addition to the above-mentioned isolates, this test included *D. eres* DiSSPA_DE1, two *N. parvum* isolates (DiSSPA_NP2 and TESAF_NP1) and *C. granati* CBS 144846 [9]. Eight and five biological replicates were used for each fungal species and control in the first and second test, respectively. Two-year-old pomegranate trees (cv. Wonderful), approximately 1.70 m in height were used. The trees were cultivated in pots measuring 25 cm in diameter and 30 cm in depth, filled with peat substrate (Terflor SRL, Capriolo, BS, Italy; pH = 6.0). These pomegranate trees were maintained under natural daylight conditions within a greenhouse shade to reduce solar radiation by 30% from April to September 2022 (first test; TESAF) and in controlled conditions (25 ± 1 °C, photoperiod 16/8, 80% RH) from July to October 2023 (second test; DiSSPA), and weekly irrigation was provided. The trees were inoculated with the pathogens at the beginning of April 2022 for the first test and at the beginning of July 2023 for the second test. Fungal isolates were inoculated as reported in [21] with slight modifications. Briefly, pomegranate stems were inoculated approximately 20 cm above the crown using a 7 mm diameter PDA agar disk excised from actively growing five-day-old mycelium (cultured in 90 mm Petri dishes containing 20 mL PDA per plate and incubated at 25 °C in the dark). The inoculation site was initially surface treated with 100% ethanol. A 10 mm diameter hole (~2 mm in depth) was made by removing the bark with a sterile scalpel, and the agar-mycelium disk was inserted into the resultant wound. To facilitate infection establishment, a cotton wool pad soaked in sterile water was applied to the inoculation site. After the inoculation, wounds were sealed with Parafilm and secured with aluminum foil. Control plants were inoculated with a sterile PDA plug using the same procedure. All trees were arranged according to a complete randomization scheme.

At six months (first test) and four months (second test) after inoculation (MAI), the bark including the site of inoculation was removed and the severity index determined according to the subsequent empiric scale based on the percentage of brown woody area (BWA) on a 20 cm stem portion (10 cm above and below the inoculation point): 0, no wood browning; 1, ≤25% of BWA; 2, 26–50% of BWA; 3, 51–75% of BWA; 4, 76–100% of BWA. The severity index was the mean value of the disease class estimated for each plant inoculated with the same pathogen. Re-isolation of the fungal species was conducted by transferring ten pieces of infected woody tissue from the margins of each lesion into PDA plates as previously described for the isolation procedure. The resulting cultures were incubated at 25 °C in the dark and subsequently identified through morphological analysis considering the shape and the color of the colony as well as the morphology of asexual and sexual sporulation structures and spores.

### 2.4. Colony Growth Under Different Temperatures and Dual Culture Assay

The colony growth of each isolate under different temperatures was assessed at 5, 10, 15, 20, 25, 30 and 35 °C in the dark. For each isolate, mycelial plugs of 4 mm in diameter were collected from the edges of five-days actively growing mycelium on MEA (90 mm diameter Petri dishes, 20 mL/plate, incubated at 25 °C under dark conditions) and used to inoculate three replicated Petri dishes (90 mm in diameter) containing the same medium. The orthogonal diameters of developing colonies were measured at 6 DAI for all isolates, and 12 DAI only for *S. stenoceras* and *Xenoacremonium* sp. (slow-growing fungi). The daily growth rate was calculated.

Dual culture assay was used to study fungal–fungal interaction (Table S2). *Diaporhte eres*, *D. foeniculina*, *N. rosae*, *S. stenocerans* and *Xenoacremonium* sp. were self-paired and cross-paired in dual culture experiments in all the inter-species combinations [32]. Additionally, we cross-paired these species with the two main pomegranate pathogens *C. granati* and *N. parvum* (DiSSPA_NP2), to assess their interactions. Three replicated Petri dishes were performed for each comparison. In dual culture plates, the radius of each colony in cross-pairing was measured, and the percentage of colony growth inhibition or promotion was calculated in comparison with the average radius of the colony in self-pairing, as reported by [32]. In some dual-culture plates, intermingling between hyphae of the two cultures was observed at 10 DAI. To assess whether fungal viability was affected, 5 mm plugs were collected from the interaction zone, and the re-isolation test was performed on MEA plates, as described by [33]. In the same assay, changes in colony color and the production of reproductive structures—conidia (*N. rosae* and *Xenoacremonium* sp.), pycnidia (*C. granati*, *D. eres*, *D. foeniculina* and *N. parvum*), and perithecia (*S. stenoceras*)—were observed at 20 DAI.

### 2.5. In Vitro Inhibition Assays with BCAs

The isolated fungi were co-cultured with three *Trichoderma* species [*T. atroviride* SC1 (Vintec; Certis Belchim B.V., Utrecht, The Netherlands) *T. asperellum* icc012 and *T. gamsii* icc080 (Tellus^®^; Syngenta Italia, Milan, Italy)] and two *Bacillus* species [*B. amyloliquefaciens* subsp. *plantarum* D747 (Amylo-X^®^; CBC Europe Srl-Biogard Division, Nova Milanese, Italy) and *B. subtilis* QST713 (Serenade Max; Bayer CropScience, Leverkusen, Germany)]. For fungal pathogens and *Trichoderma* species, a 7 mm diameter MEA agar disk, taken from five-days actively growing mycelium (90 mm diameter Petri dishes, 20 mL/plate, incubated at 25 °C under dark conditions) was placed on 90 mm MEA plates at 10 mm from the border of the plate. For *Bacillus* species, bacterial cells were collected from two day-old nutrient agar [NA; 0.8% Nutrient broth (Merck, Darmstadt, Germany), 2% European bacteriological agar (LLG labware)] cultures, and resuspended in sterile distilled water. Cells suspensions were taken up to OD = 0.1 (~1 × 10^8^ CFU mL^−1^) by using a spectrophotometer (DU-640; Beckman, Fullerton, CA, USA) and a 5 µL drop was spotted at 10 mm from the border of the plate. Dual culture plates were incubated at 25 ± 1 °C and monitored every two days up to 10 DAI. Three replicated Petri dishes were performed for each treatment. The radius of each colony (pathogen and BCA) in the co-inoculation plates was measured and the percentage of colony growth inhibition of each fungal pathogen was calculated in comparison with the radius of the colony in control plates (pathogen grown alone), as reported by [32].

### 2.6. Statistical Analysis

Differences among isolates in the pathogenicity assay (severity index), colony growth in fungal–fungal combinations in dual culture assays, and colony growth inhibition by *Bacillus* and *Trichoderma* isolates were analyzed by one-way ANOVA followed by Tukey’s HSD test at *p* = 0.05. The analyses were conducted in CoStat (version 4.451; CoHort Software, Monterey, CA, USA). Daily growth rate data under different temperatures were used to study the relationship among isolates with the correlation-based clustering in Minitab software (version 19.2020.1) (www.minitab.com).

## 3. Results

### 3.1. Field Inspections, Symptom Description and Pathogen Isolation

**Figure 1:**
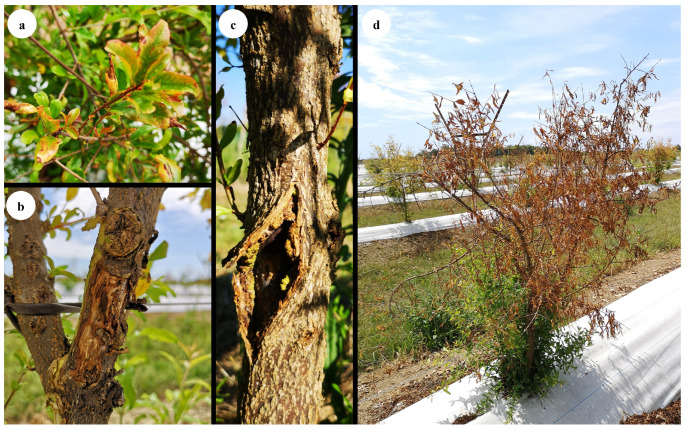
Disease symptoms detected on pomegranate trees in field surveys. Leaves showing chlorosis, reddish margins and necrosis (**a**), branch canker (**b**), stem canker (**c**) dieback and death symptoms (**d**).

From 2016 to 2020, trees showing dieback symptoms were observed in four pomegranate orchards: one each in Apulia and Basilicata (July 2016 and September 2017) and two in Veneto region (July 2020), Italy. In all orchards, pomegranate trees showed a complex symptomatology. In Apulia and Basilicata sites, the examined trees showed dieback, necrotic lesions and the presence of cankers at the branch level. In both Veneto sites pomegranate trees exhibited a similar symptomatology, i.e., stunted growth, stem and branch cankers, diebacks, leaf chlorosis and necrosis, general decline and death symptoms (Figure 1). In Veneto region, pomegranate trees of the San Biagio di Callalta site exhibited more advanced dieback symptoms as compared to the Legnaro site. At all sites, no symptoms were detected in the roots, which appeared healthy. The prevalence of symptomatic plants was 10% and 15% in the orchards located in Apulia and Basilicata, respectively, and 25% and 30% in the Veneto orchards located in Legnaro and San Biagio di Callalta, respectively.

In symptomatic trees, internal wood browning was exclusively detected in branch and stem samples. In the orchards located in the Apulia and Basilicata regions, wood browning was limited to some branches or stems within each tree. Specifically, seven affected branches and one affected stem were collected from the orchard in Apulia, while five symptomatic branches were obtained from the orchard in Basilicata. In contrast, in the symptomatic trees examined in both orchards of Veneto region, all branches and stems displayed internal wood browning; therefore, a total of nine branches and three stems per orchard were analyzed. The details of the fungal morphologies and the total number of fungal isolates recovered from pomegranate trees and their abundance are shown in Table 1. From the symptomatic branch samples of pomegranate trees collected in the Apulia orchard, *Diaporthe*-like and *N. parvum* morphology isolates were obtained. Isolates with *Diaporthe*-like morphology were obtained from one pomegranate tree, specifically from the only branch sample that exhibited internal wood browning. From each of the remaining two trees, both *Diaporthe*-like and *N. parvum* morphologies were recovered from branch samples. In one pomegranate tree, *N. parvum* was also isolated from the stem. In the orchard located in Basilicata region only *N. parvum* was obtained from branch samples, while stem samples appeared healthy. From the symptomatic branch samples of pomegranate trees collected in San Biagio di Callalta, *Diaporthe*-like, *Neopestalotiopsis*-like and *N. parvum* morphology isolates were obtained. Isolates with *Diaporthe*-like and *N. parvum* morphology were isolated from the same tree. Finally, in the Legnaro site, isolates with *Neopestalotiopsis*-like, *Sporothrix*-like and *Xenoacremonium*-like morphology were obtained. Isolates with *Neopestalotiopsis*-like and *N. parvum* morphology were obtained from different branches of the same tree. Isolates with *Sporothrix*-like and *Xenoacremonium*-like morphology were obtained from pomegranate stems with canker lesions (Table 1).

Except for *N. parvum* and the *Diaporthe*-like isolates, the morphologies obtained in this study correspond to fungal genera not previously reported on pomegranate as causal agents of dieback. For each of these morphotypes, one representative isolate was selected for further characterization.

### 3.2. Morphological Identification

**Figure 2:**
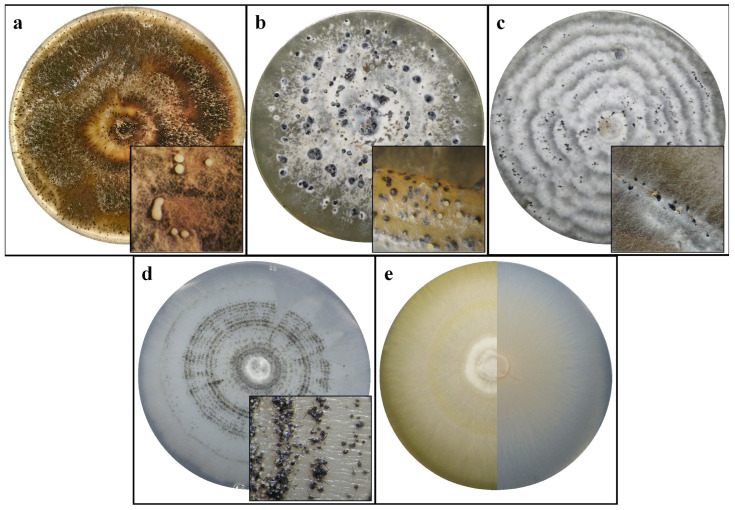
In vitro growth of *Diaporthe eres* (**a**), *D. foeniculina* (**b**), *Neopestalotiopsis rosae* (**c**), *Sporothrix stenoceras* (**d**) and *Xenoacremonium* sp. (**e**) after 14 days of incubation. For *Xenoacremonium* sp. colony morphology on malt extract agar (MEA; left) and potato dextrose agar (PDA; right) is shown. For the other fungal pathogens, the colonies grown on MEA are shown. The close views of *D. eres* and *D. foeniculina* show pycnidia developed on MEA and Water Agar-fennel, respectively. The close views of *N. rosae* and *S. stenoceras* show acervuli and perithecia developed on Water Agar needles and PDA, respectively. Close views were taken under a stereomicroscope.

**Figure 3:**
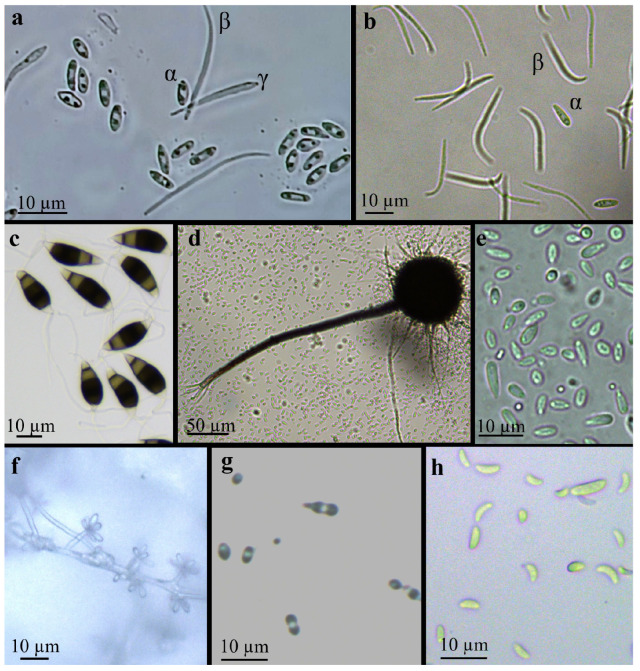
α, β and γ conidia of *Diaporthe eres* (**a**), α and β conidia of *D. foeniculina* (**b**), conidia of *Neopestalotiopsis rosae* (**c**), perithecium (**d**), ascospores (**e**), conidiophores (**f**), conidia (**g**) of *Sporothrix stenoceras* and conidia of *Xenoacremonium* sp. (**h**).

The isolate with *Diaporthe*-like morphology obtained from the Basilicata orchard produced an aerial, white mycelium that turned brown after about seven days. Abundant black pycnidia were produced after ten days (Figure 2a). Conidia α or β were frequently observed, while in some cases γ conidia were also observed. The α conidia were unicellular, hyaline, biguttulate, and measured 5.3–6.5 µm × 2.3–3.5 µm, (average 6.0 × 2.6 µm), the β conidia were filiform, hyaline and measured 0.7–1.1 × 16.3–29.4 µm (average 1.1 × 23.1 µm), while the γ conidia were hyaline, multiguttulate, fusiform to subcylindrical and measured 12.0–16.3 × 2.0–3.3 µm (average 14.6 × 2.5 µm) (Figure 3a). These features addressed the identification toward *Diaporthe eres* [34] and the isolate was designated as DiSSPA_DE1. The daily growth rate ranged from 10.1 mm/day (WA) to 18.0 mm/day (PDA) (Table 2).

The isolate exhibiting *Diaporthe*-like morphology obtained from San Biagio di Callalta (Veneto) produced white cottony mycelium and differentiated dark pycnidia after seven days (Figure 2b). On WA-fennel, pycnidia were produced in greater abundance than on the other media. Unlike the *Diaporthe* isolate from Basilicata, the Veneto isolate produced only α and β conidia. The α conidia were unicellular, hyaline, ovoid or fusiform, measuring 6.2–18.5 × 2.0–3.9 µm (average 9.11 × 2.85 µm) (Figure 3b), while the β conidia, more abundant, were unicellular, hyaline, filiform, aseptate, slightly curved and measuring 18.3–42.4 × 1.8–2.0 µm (average 31 × 2 µm) (Figure 3b). These characteristics were consistent with *Diaporthe foeniculina* [35] and the isolate was designated TESAF_DF1. The daily growth rate ranged from 5.1 mm/day (WA) to 15.9 mm/day (WA-fennel) (Table 2).

The isolate with *Neopestalotiopsis*-like morphology produced white cottony mycelium, with the emergence of acervuli at seven days on WA-pine needles and within 28 days on the other media (Figure 2c). The conidia were ellipsoidal, consisting of five cells, with the two apical cells being hyaline and the three central cells being dark. These conidia measured 22.0–30.5 × 6.5–11.9 µm (average 25.3 × 8.6 µm) and presented three apical appendages (occasionally four) and one shorter basal appendage (Figure 3c). These characteristics were consistent with *Neopestalotiopsis rosae* [36] and the isolate was designated TESAF_NR1. The average daily growth rate was higher on OA and WA-pine needles (11.2 mm/day) with respect to PDA, MEA and WA (Table 2).

The isolate with *Sporothrix*-like morphology developed hyaline mycelium on WA, light luteal on MEA, and white on the other media. Dark perithecia were arranged in concentric circles, superficially or partially immersed in the media (Figure 2d). These structures were characterized by a globose base ornamented with brown hyphal hair and a neck straight or slight curved (Figure 3d). They were 132–598 µm long (average 413.6 µm) and were observed on all media within seven days with the exception of WA (Figure 2d). Perithecia reached maturity after approximately 14 days, developing ostiolar hyphae at their terminal ends. The ascospores were unicellular, hyaline, and allantoid, measuring 3.0–8.7 × 1.6–3.8 µm (average 5.3 × 2.5 µm) (Figure 3e). Conidia (Figure 3f) were borne at the apex of hyaline conidiophores (Figure 3g) and measured 3.3–7.4 × 0.8–1.6 µm (average 5.6 × 1.2 µm). These characteristics were consistent with the *Sporothrix stenoceras* species complex, and specifically the isolate was assigned to *S. stenoceras* [37,38] and designated TESAF_SS1. The daily growth rate of *S. stenoceras* was within the range of 2.7 to 3 mm/day across all media (Table 2).

Finally, the isolate with *Xenoacremonium*-like morphology produced mycelium partially embedded in the medium. In the oldest part of the colony, the mycelium developed distinct colorations: pink on MEA and ochre on PDA (Figure 2e). Conidia appeared after 28 days on WA and 14 days on the other media. They were unicellular, hyaline, and falciform, measuring 3.6–16.6 × 1–1.4 µm (average 6.5 × 1 µm) (Figure 3h). These features correspond to the genus *Xenoacremonium* [39]. Morphological analysis alone was not sufficient to achieve species-level identification. The isolate was then designated TESAF_X1. The average daily growth rate was 2.9 mm/day on PDA and 3.2, mm/day on MEA, OA and WA (Table 2).

### 3.3. Molecular Identification

Sequences of the selected isolates used in the BLASTn analysis have been deposited in NCBI. Their accession numbers, along with those of the isolates included in the phylogenetic analysis, are provided in Tables S3 and S4, respectively. BLASTn analysis allowed species-level identification for five out of six morphologies, with >99% identity observed when each sequence was compared against the “core_nt” database, which limits the analysis to the type strain sequences. For all fungal isolates, the BLASTn-based molecular identification confirmed the morphological one. For the *Xenoacremonium*-like morphology, the BLASTn analysis of ITS and LSU sequences resulted in >99% identity with the ITS and LSU sequences of the *Xenoacremonium falcatum* CBS 400.85 and *X. recifei* CBS 137.35 (type strains). Conversely, the BLASTn analysis of the *TEF1*, *cmdA* and *TUB2* sequences resulted in identities of 94%, 97% and 95%, respectively, with *X. falcatum* CBS 400.85, and 91%, 92% and 95% with *X. recifei* CBS 137.35. When the BLASTn search was extended to the full core_nt database, the *TEF1* sequence showed 99.5% identity (41% coverage) with *X. minutisporium* KNUF-20-047 (OP515525.1), while *cmdA* and *TUB2* sequences continued to show low identity values (<97%) with different isolates of *X. falcatum* and *X. recifei*. Therefore, the BLASTn analysis does not allow us to identify the *Xenoacremonium* isolate at species level.

The BLASTn-identification was confirmed by the ML phylogenetic analysis (Figure S1). The phylogenetic analysis based on the combined ITS-*TEF1*-*TUB2* sequences of the *Diaporthe* species resulted in a tree with the highest log-likelihood value of −9101.43. According to this analysis, *D. eres* DiSSPA_DE1 and *D. foeniculina* TESAF_DF1 grouped with the respective type strains AR5193 (100%) and CBS 111553 (99%) (Figure S1a). Within the *eres* section, the species closest related to *D. eres* were those of the *D. gardeniae* and *D. citrichinensis* species complexes. *Diaporthe chamaeropsis*, *D. parvae* and *D. forlicesenica* were the species most closely related to *D. foeniculina* (Figure S1a). According to the tree obtained with ITS-*TEF1*-*TUB2* sequences of the *Neopestalotiopsis* species (highest log-likelihood value: −2832.33), *N. roase* TESAF_NR1 grouped with the type strain *N. rosae* CBS 101057, and the most closely related species were *N. javanensis*, *N. maldoxii* and *N. celtidis* (Figure S1b). Using the combined ITS-LSU-*TUB2* sequences of the *Sporothrix* species, we obtained a tree with the highest log-likelihood value of −4501.15. According to this analysis, *S. stenoceras* TESAF_SS1 clustered with its type species CMW 3201 (100%) and the most closely related species was *S. narcissi* (Figure S1c). Finally, according to the tree obtained with combined ITS-LSU-*TEF1* sequences of *Xenoacremonium* species (highest log-likelihood value: −4481.61), the TESAF_X1 isolate clustered with *Xenoacremonium* sp. KNUF 20-047. *X. falcatum* was the most closely related species (99%) with *X. allantoideum* and *X. recifei* representing another group (98%) (Figure S1d).

Considering both morphological and molecular identification, the occurrence of fungal isolates in the monitored orchards was determined. *Neofusicoccum parvum* was isolated in all four monitored sites. *Diaporthe eres* was detected in Apulia. *Neopestalotiopsis rosae* was isolated from both sites of Veneto region. *Diaporthe foeniculina* was isolated only in Veneto from the San Biagio di Callalta orchard. *Xenoacremonium* sp. and *S. stenoceras* were found in Legnaro site of Veneto region.

### 3.4. Pathogenicity Test

In both pathogenicity tests that were conducted, all the fungal species produced necrotic lesions that spread up and down from the inoculation site. In the control tree, small necrotic lesions were limited to the site of inoculation (Figure S2). Table 3 presents the data of severity index and re-isolation percentage for each fungal isolate. In the first pathogenicity test, the severity index ranged between 1.8 and 2.8 for all fungal species, significantly higher than the control whose severity was <1. For *N. rosae*, the severity was 2.8 and it was statistically different from that observed for *S. stenoceras* (1.8). The percentage of re-isolation was 100% for *N. rosae* and *D. foeniculina*, and 88% for *S. stenoceras* and *Xenoacremonium* sp. In the second pathogenicity test, the severity index of the selected fungal isolates ranged between 2.4 and 3, showing statistically significant differences compared with the control, whose severity index was 0.4 ± 0.2. The fungal species were re-isolated from the inoculated plants with a percentage included in the range 56% (*N. rosae*) to 96% (*N. parvum*).

### 3.5. Colony Growth Under Different Temperatures and Fungal-Fungal Interactions

**Figure 4:**
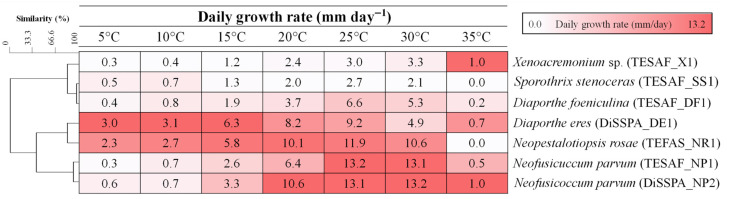
Daily growth rate on malt extract agar at different temperatures of the fungal pathogens obtained from pomegranate plants showing dieback symptoms. The dendrogram was generated using correlation-based clustering in Minitab software (www.minitab.com) to assess the percentage similarity among fungal isolates.

Fungal daily growth rates were measured on MEA at various temperatures and analyzed using correlation-based clustering (Figure 4). *N. parvum* isolates clustered together (similarity = 94%) and exhibited the highest daily growth rates (13.1–13.2 mm/day) at 25 and 30 °C. *Diaporthe eres* and *N. rosae* showed similar behavior (81.3% similarity), with the highest daily growth rates at lower temperatures (5–15 °C). *Diaporthe foeniculina*, *S. stenoceras* and *Xenoacremonium* sp. (81.2% similarity) showed low growth rates at 15 and 20 °C compared to the other species. At 20, 25 and 30 °C, *D. foeniculina* grew faster than *S. stenoceras* and *Xenoacremonium* sp. The optimum temperature was 25 °C for all fungi, except for *Xenoacremonium* sp., which exhibited an optimum at 30 °C. At 35 °C the growth of *S. stenoceras* and *N. rosae* was completely inhibited, while a residual growth was detected in the remaining fungi.

**Figure 5:**
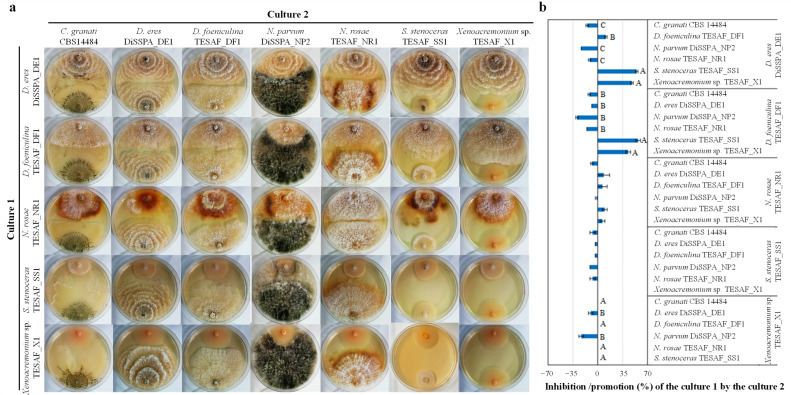
Dual culture assay (**a**) and percentage of colony growth inhibition or promotion (**b**) of *Diaporthe eres*, *D. foeniculina*, *Neopastalotiopsis rosae*, *Sporothrix stenoceras* and *Xenoacremonium* sp. when co-cultured with each other or with *Coniella granati* and *Neofusicoccum parvum*. Data are reported as the average ± standard error. For each pathogen, differences in terms of colony growth, and promotion or inhibition in co-culture were assessed using Tukey’s test at a significance level of 0.01 (uppercase letters).

Given the observed co-occurrence of some of the isolated pathogens within the same tree (*N. parvum* was isolated together with *D. eres* in Apulia, and with *D. foeniculina* or *N. rosae* at the Veneto sites of Legnaro and San Biagio di Callalta, respectively), interspecific interactions among all the newly identified pomegranate pathogens were investigated using dual culture assays. In addition to the ubiquitous *N. parvum*, *C. granati*, one of the main pathogens of pomegranate was included in the assay. The test was conducted at 25 ± 1 °C. Growth, colony color and production of reproductive structures were evaluated for each isolate in self-pairing and cross-pairing with the other pathogens at 10 (Figure 5a) and 20 DAI. *N. rosae* produced white mycelium in self-pairing and brown colonies in dual culture with other fungi. At 20 DAI, *D. eres* exhibited brownish coloration in self-pairing and in combination with *D. foeniculina*, while colonies remained white in all other combinations. *Diaporthe foeniculina* turned brown only when co-cultured with *N. rosae*. *Sporothrix stenoceras* showed differences in perithecia production at 20 DAI with limited production when co-cultured with *N. rosae* and extensive production (>50% of the colony surface) when paired with *D. eres* and *Xenoacremonium* sp. *Xenoacremonium* sp. maintained consistent colony morphology across all tested pairings.

The effects of co-culturing on fungal growth are presented in Figure 5b. *Diaporthe eres* and *D. foeniculina* showed a significant increased growth when co-cultured with *S. stenoceras* and *Xenoacremonium* sp. (slow-growing fungi) and reduced growth when paired with *C. granati*, *N. rosae* and mostly *N. parvum. Neopestalotiopsis rosae* colony growth was weakly affected by the other fungi compared to self-pairing. *Sporothrix stenoceras* growth was slightly inhibited by all fungal species except *Xenoacremonium* sp. This latter was inhibited only by *D. eres* and *N. parvum*.

When the growing fungal colonies came into proximity, their growth was generally arrested at the point of contact. Mutual compatibility between hyphae was observed at 20 DAI in the combinations *D. foeniculina*/*S. stenoceras*, *N. rosae*/*Xenoacremonium* sp. and *Xenoacremonium* sp./*C. granati*. For each combination, both fungal isolates were successfully re-isolated from the contact area. Similar behavior was observed at 10 DAI for combinations of *N. parvum* with *D. foeniculina*, *D. eres*, *Xenoacremonium* sp. and *S. stenoceras*. *Neofusicoccum parvum* mycelium exhibited varying degrees of overgrowth on the other fungal isolates, ranging from 22% (*D. foeniculina*) to 100% (*S. stenoceras*). Despite complete overgrowth, *S. stenoceras* remained viable in the presence of *N. parvum* as both pathogens were successfully re-isolated from the overgrown mycelial plugs.

### 3.6. In Vitro Effectiveness of Bacillus and Trichoderma BCAs

**Figure 6:**
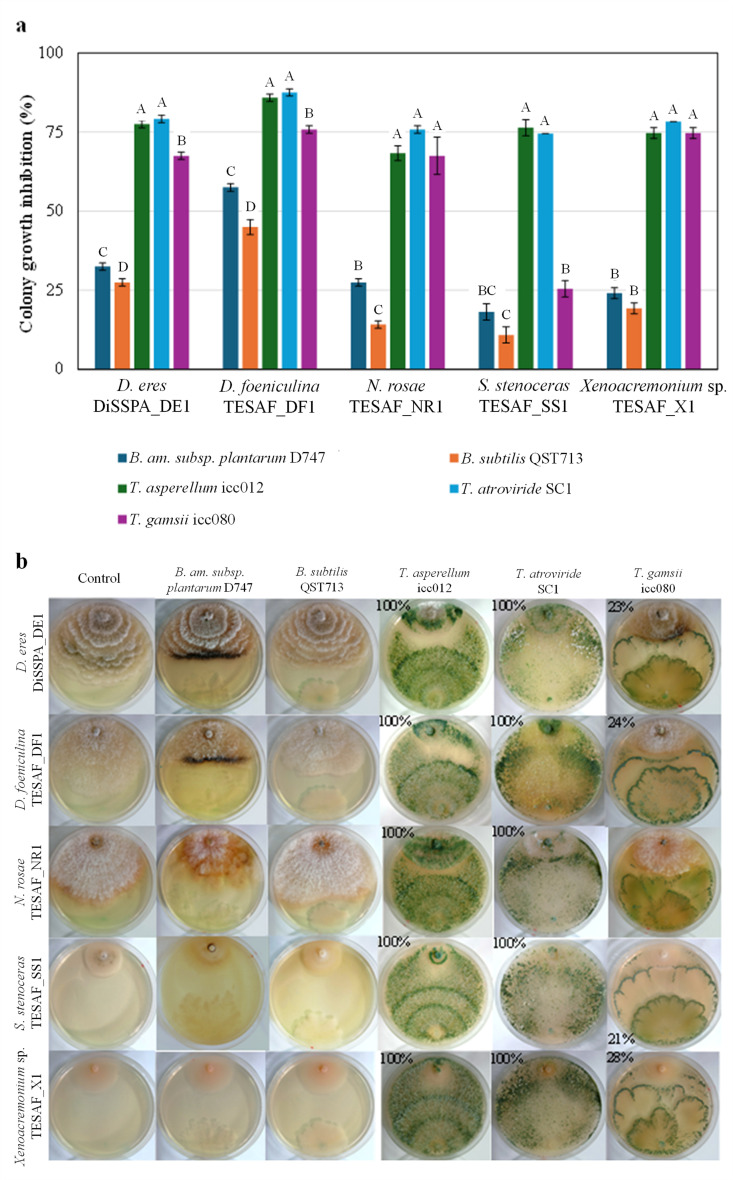
Colony growth inhibition (**a**) by *Bacillus amyloliquefaciens* subsp. *plantarum*, *B. subtilis*, *Trichoderma asperellum*, *T. atroviride* and *T. gamsii* against *Diaporthe eres*, *D. foeniculina*, *Neopestalotiopsis rosae*, *Sporothrix stenoceras* and *Xenoacremonium* sp. in dual culture assay (**b**). Data are reported as the average ± standard error. The value shown in each image represents the overgrowth percentage of *Trichoderma* species over the colony of each target fungus (when reported in the upper part of the colony image) or of the target fungus over the *Trichoderma* colony (when reported in the lower part of the colony image, only in the *S. stenoceras*/*T. gamsii* combination). For each pathogen, significant differences in colony growth-inhibition percentages caused by *Bacillus* and *Trichoderma* isolates were determined using Tukey’s test at a significance level of 0.01 (uppercase letters).

The efficacy of commercial plant protection products containing *Bacillus* and *Trichoderma* species was evaluated using dual culture assays (Figure 6). Among the two bacterial species, *B. amyloliquefaciens* demonstrated significantly higher activity compared to *B. subtilis*. *Diaporthe foeniculina* showed the highest sensitivity to *Bacillus* with growth reduction of 57.5% and 45.0% by *B. amyloliquefaciens* and *B. subtilis*, respectively. For all the other *Bacillus*–pathogen combinations, growth reduction was below 30% (Figure 6a). *Sporothrix stenoceras* exhibited the lowest sensitivity to *Bacillus* with inhibition rates of 18% and 10% for *B. amyloliquefaciens* and *B. subtilis*, respectively. An inhibition halo was observed in all *Bacillus*–pathogen combinations.

Among the three *Trichoderma* species, *T. asperellum* and *T. atroviride* showed statistically comparable efficacy against all pathogens, while *T. gamsii* was significantly less active against *D. eres*, *D. foeniculina* and *S. stenoceras*. All three *Trichoderma* species demonstrated similar activity against *N. roseae* and *Xenoacremonium* sp. Inhibition values were close to or higher than 70% in most combinations. The only exception was *T. gamsii*, which showed only 25% inhibition in combination with *S. stenoceras* (Figure 6a). Ten days after co-inoculation, *T. atroviride* and *T. asperellum* overgrew the mycelium of pomegranate pathogens and produced abundant sporulation. In contrast, *T. gamsii* exhibited limited overgrowth, covering only about 25% of *D. eres*, *D. foeniculina*, and *Xenoacremonium* sp. colonies. No *T. gamsii* growth was observed over the *N. rosae* colony. Notably, when cultured with *S. stenoceras*, the pathogen visibly grew over *T. gamsii* (Figure 6b).

## 4. Discussion

Pomegranate dieback is a disease with a complex etiology that remains only partially understood. This study advances knowledge of the disease by identifying and characterizing both previously reported and newly identified pomegranate pathogens. Among these, the two *Diaporthe* species detected were *D. eres* and *D. foeniculina*, which have also been isolated from pomegranate with wood decay symptoms in Lazio, Italy [40]. Both species are well known as causal agents of stem, branch and shoot cankers on various fruit crops worldwide, including in Italy, and have been reported to cause Phomopsis cane disease on grapevine, together with *D. ampelina* [41,42,43].

*Neofusicoccum parvum* is reported here for the first time in pomegranate in southern Italy (Basilicata and Apulia). We observed the co-occurrence of *N. parvum* and *D. eres*. Similarly, this association was reported by [40], suggesting a potential synergistic role in symptom development. This study associates for the first time *N. rosae*, *S. stenoceras*, and *Xenoacremonium* sp. with pomegranate dieback. *Neopestalotiopsis rosae* was isolated from both sites in Veneto, indicating it may play a more prominent role in pomegranate dieback than *S. stenoceras*, and *Xenoacremonium* sp., both of which were isolated only once during the study. However, the low isolation frequency of *S. stenoceras* and *Xenoacremonium* sp. may be attributed to their slow in vitro growth, which could have affected the isolation process. All species were identified morphologically and molecularly, based on previous studies describing them [34,35,36,37,38,39], and pathogenicity assays demonstrated their capacity to produce wood browning on pomegranate trees. Severity index data were consistent between the two pathogenicity experiments, with slight differences for *S. stenoceras* and *Xenoacremonium* sp. likely attributable to the different experimental conditions.

*Neopestalotiopsis rosae* has been previously reported as responsible for foliar and fruit spots on pomegranate in the USA [44]. Here we report its ability to produce wood symptoms in pomegranate. Species belonging to the *Neopestalotiopsis* genus are widely distributed in temperate areas and occur as plant pathogens on both herbaceous and woody hosts, causing stem-end rot, dieback and trunk diseases [45]. Several *Neopestalotiopsis* species, including *N. rosae*, have recently been identified as emerging fungal pathogens in Italy. Examples include *N. siciliana*, associated with dieback in avocado plants [46,47]; *N. hispanica*, linked to dieback in bottlebrush and myrtle [48]; and several other species implicated in crown rot of strawberry [49].

*Sporothrix stenoceras* (syn. = *Ophiostoma stenoceras*), identified here for the first time in association with pomegranate dieback, is well known as a wood-colonizing fungus on coniferous and hardwood hosts [50,51]. Other Ophiostomatoid fungi (e.g., *O. ulmi*, *O. novo-ulmi*, *O. quercus*) are associated with severe diseases that pose major threats to elm, eucalyptus and oaks [52,53].

The *Xenoacremonium* isolate obtained in this study was difficult to identify at the species level. The genus *Xenoacremonium* currently includes six species, *X. allantoideum*, *X. brunneosporum*, *X. falcatum*, *X. minutisporum*, *X. palmarum* and *X. recifei* (https://indexfungorum.org/, accessed on 15 October 2025). Of these, *X. falcatum* and *X. palmarum* have been reported in association with pomegranate fruit rot and date palm root rot in Iran, respectively [54,55], while *X. brunneosporum* has been isolated from the cortex of *Rhizophora mucronate* [56]. The other species have been isolated from soil and insects [57,58]. Our isolate was morphologically similar to *X. falcatum* [54,57,58] but molecular and morphological analyses did not allow species-level identification. Based on the currently available morphological and molecular data, the *Xenoacremonium* isolate obtained from pomegranate trees showing dieback may represent a new species. Future studies using additional genetic markers and phylogenetic informative genes will clarify the taxonomy of our *Xenoacremonium* isolate.

The environment plays a crucial role in shaping the dynamics of pathogens and microbial communities, particularly in the context of ongoing climate change, which is driving the emergence of new diseases [59,60]. To explore this aspect, we evaluated colony growth at different temperatures. For all isolates, except *D. eres* (20–25 °C), the optimal growth temperature ranged between 25 °C and 30 °C. At both temperatures, *N. parvum* exhibited the fastest growth among all fungal species. This result is consistent with previous studies assessing *N. parvum* growth under in vitro and in vivo conditions [61,62]. At 5–10 °C, *D. eres* and *N. rosae* showed the highest growth rates, suggesting their potential to cause disease symptoms even at low temperatures, although the optimal temperature for growth may differ from that for pathogenicity. While a previous study reported the ability of *N. rosae* to grow rapidly at mild temperatures, causing stem lesions and dieback on avocado in Italy [46], our results indicate that this species can also sustain significant growth at even lower temperatures.

Among the isolated pathogens, *S. stenoceras* and *Xenoacremonium* sp. exhibited slow growth. For both fungi, the growth rate is reported here for the first time, and the detected values were comparable to those of others well-characterized pomegranate plant pathogens, such as *Phaeoacremonium* spp. and *Ceratocystis fimbriata* [63,64]. When artificially inoculated on pomegranate trees, *Xenoacremonium* sp. caused higher disease severity than *S. stenoceras*, comparable to that produced by *C. granati* and *N. parvum*. These results highlight a discrepancy between the slow in vitro growth of *Xenoacremonium* sp. and its high disease severity, supporting the notion that in vitro growth rates do not necessarily correlate with pathogenicity.

In vitro cross-pairing is generally the first approach to study fungus–fungus interactions [65]. In this study, *N. parvum* was found in the same tree with *D. eres* in Apulia, with *D. foeniculina* and with *N. rosae* in Veneto sites of Legnaro and San Biagio di Callalta, respectively. Accordingly, in vitro cross-pairing assays demonstrated that *N. parvum* does not devitalize the other pathogens when grown in co-culture. Overall, the interaction observed in the cross-pairing assays was mainly driven by competition, since the colony growth of the target fungi was reduced when grown in cross-pairing with faster isolates like *N. parvum* and *N. rosae* as compared to self-pairing. Among the other fungal pathogens, *N. rosae* produced white mycelium in self-pairing, turning brown when grown in dual culture with other fungi. This effect was more pronounced when paired with *D. eres* and less evident with *S. stenoceras*, suggesting that metabolite production could depend on the co-cultured species, as observed in other fungus–fungus cross-pairings [66].

The production of perithecia by *S. stenoceras* in cross-pairing with *N. rosae*, *D. eres*, and *Xenoacremonium* sp. could be interpreted as a stress-induced response, potentially triggered by the presence of competing species; fungi exhibit multiple strategies to survive under stress conditions [67]. Notably, *S. stenoceras* displayed variation in perithecia production depending on the pairing partner, with limited formation observed in co-culture with *N. rosae* and extensive production when paired with *D. eres* and *Xenoacremonium* sp., suggesting that the degree of perithecia production may correlate with the intensity of the competitive interaction. For the cross-pairing combinations *D. foeniculina*/*S. stenoceras*, *D. foeniculina*/*Xenoacremonium* sp., *N. rosae*/*Xenoacremonium* sp. and *Xenoacremonium* sp./*C. granati*, as well as for all fungal pathogens in cross-pairing with *N. parvum* except *N. rosae*, intermingling between hyphae was observed, suggesting potential mutualistic interactions. Further research in vivo, including the host plant, will help to clarify possible synergistic effects on pomegranate dieback caused by these pathogens, a feature already reported for *C. granati* and *P. palmivora* under drought and waterlogging as abiotic stressors [21]. Future studies should also quantify the economic impact of pomegranate dieback in Italian orchards.

The occurrence of new pathogenic species on pomegranate represents an important challenge for its phytosanitary management, considering that according to the current regulations, alternative control measures to synthetic fungicides should be preferred to ensure sustainable agriculture [68]. *Bacillus* and *Trichoderma* include species that have been extensively studied for application as BCAs in pomegranate protection [21,69]. Dual-culture assays were used to evaluate the antagonistic potential of *Bacillus* and *Trichoderma* BCAs already included in commercial formulations registered for the management of fungal pathogens in other crops [70,71,72,73]. Both *Trichoderma* and *Bacillus* BCAs reduced the colony growth of all fungal pathogens, with some variations depending on the specific BCA–pathogen combination. For the *Trichoderma* species tested, particularly *T. asperellum* and *T. atroviride*, competition and hyperparasitism appeared to be the primary modes of action, as these fungi exhibited rapid growth and were able to overgrow the pathogens [74,75]. Conversely, when the pomegranate pathogens were grown with *Bacillus* BCAs, an inhibition halo was observed, indicating that antibiosis was the primary mode of action responsible for suppressing pathogen growth. Both *B. amyloliquefaciens* subsp. *plantarum* D747 and *B. subtilis* QST713 are well known for their ability to produce non-ribosomal peptides responsible for antibiotic effects [76]. However, antibiosis is also a well-known trait of *Trichoderma* species [74]. Further studies under field conditions are required to identify effective protection strategies that incorporate these biological approaches. This aspect is of particular interest, as *Trichoderma* and *Bacillus* BCAs are already included in plant protection products registered for use on pomegranate and other fruit trees.

This study highlights the need to implement phytosanitary monitoring programs, contributes to a better understanding of pomegranate dieback etiology and expands the spectrum of pathogens that can be targeted in future management strategies, ultimately benefiting pomegranate growers.

## 5. Conclusions

In conclusion, this work reports fungal pathogens as causal agents of dieback on pomegranate. Among them, *N. parvum* has been previously reported, while *D. eres* and *D. foeniculina* were recently associated with pomegranate wood decay [40]. Notably, *N. rosae*, *S. stenoceras*, and *Xenoacremonium* sp. are reported here for the first time in pomegranate showing dieback symptoms. All pathogens exhibited optimal growth between 25 and 30 °C, with *D. eres* and *N. rosae* also showing adaptability to lower temperatures. Dual culture assays showed that these fungi can share the same ecological niche while maintaining their viability. Finally, both *Bacillus* and *Trichoderma* BCAs, already included in registered crop protection formulations, appear promising for the control of these newly identified fungal pathogens.

## Figures and Tables

**Table 1:** Fungal isolates obtained from pomegranate trees showing dieback symptoms.

Region	Symptomatic Samples	Morphotype	
		Description ^b^	Frequency of Isolation ^c^
Apulia	Branch	*Diaporthe*-like	3/3
	Branch and stem	*Neofusicoccum parvum*	2/3
Basilicata	Branch	*Neofusicoccum parvum*	3/3
Veneto (Site 1) ^a^	Branch	*Diaporthe*-like	2/3
	Branch and stem	*Neofusicoccum parvum*	2/3
	Branch	*Neopestalotiopsis*-like	2/3
Veneto (Site 2) ^a^	Branch	*Neofusicoccum parvum*	1/3
	Branch	*Neopestalotiopsis*-like	1/3
	Stem	*Sporothrix*-like	1/3
	Stem	*Xenoacremonium*-like	1/3

^a^: Site 1 in Veneto region is located in the countryside of San Biagio di Callalta (Treviso province) while Site 2 is located in the countryside of Legnaro (Padova province). ^b^: The morphology was assigned to the corresponding species or genus based on colony characteristics, as well as on the features of asexual and sexual reproductive structures and spores. ^c^: Number of symptomatic trees from which each morphotype was isolated.

**Table 2:** Daily growth rates of the fungal isolates under study in different media.

Fungal Isolate	Media	Daily Growth Rate (mm/Day) *
*Diaphorthe eres* DiSSPA_DE1	Water agar	10.1 ± 0.1
	Potato dextrose agar	18.0 ± 0.3
	Malt extract agar	12.3 ± 0.1
	Oatmeal agar	15.0 ± 0.0
	Water agar-fennel	14.5 ± 0.5
*Diaporthe foeniculina* TESAF_DF1	Water agar	5.1 ± 0.2
	Potato dextrose agar	6.9 ± 0.1
	Malt extract agar	8.9 ± 0.1
	Oatmeal agar	10.7 ± 0.2
	Water agar-fennel	15.9 ± 0.6
*Neopestalotiopsis rosae* TESAF_NR1	Water agar	7.9 ± 0.1
	Potato dextrose agar	9.5 ± 0.2
	Malt extract agar	8.2 ± 0.3
	Oatmeal agar	11.2 ± 0.3
	Water agar-pine needles	11.2 ± 0.3
*Sporothrix stenoceras* TESAF_SS1	Water agar	2.8 ± 0.1
	Potato dextrose agar	3.0 ± 0.1
	Malt extract agar	2.7 ± 0.1
	Oatmeal agar	2.7 ± 0.1
*Xenoacremonium* sp. TESAF_X1	Water agar	3.2 ± 0.1
	Potato dextrose agar	2.9 ± 0.0
	Malt extract agar	3.2 ± 0.1
	Oatmeal agar	3.2 ± 0.1

* Daily growth rate was calculated at 4 days after inoculation. Data are the average of three replicates ± standard error.

**Table 3:** Severity index and re-isolation percentage observed in the pathogenicity assays for the selected fungal isolates obtained from symptomatic pomegranate trees.

Fungal Isolate	First Test (TESAF) *	Second Test (DiSSPA) *
Control	0.8 ± 0.5 c (0)	0.4 ± 0.2 b (0)
*Coniella granati* (CBS 144846)	-	3.0 ± 0.3 a (76)
*Diaporthe eres* (DiSPPA_DE1)	-	2.4 ± 0.4 a (88)
*Diaporthe foeniculina* (TESAF_DF1)	2.4 ± 0.4 ab (100)	2.4 ± 0.2 a (74)
*Neopestalotiopsis rosae* (TESAF_NR1)	2.8 ± 0.9 a (100)	2.4 ± 0.2 a (56)
*Neofusicoccum parvum* (DiSSPA_NP2)	-	3.4 ± 0.2 a (92)
*Neofusicoccum parvum* (TESAF_NP1)	-	3.0 ± 0.4 a (96)
*Sporothrix stenoceras* (TESAF_SS1)	1.8 ± 0.5 b (88)	2.4 ± 0.4 a (68)
*Xenoacremonium* sp. (TESAF_X1)	2.2 ± 0.7 ab (88)	3.0 ± 0.4 a (92)

* Severity index determined according to the subsequent empiric scale based on the percentage of brown woody area (BWA) on a 20 cm stem portion (10 cm above and below the inoculation point): 0, no wood browning; 1, ≤25% of BWA; 2, 26–50% of BWA; 3, 51–75% of BWA; 4, 76–100% of BWA. Data are the average of eight (first test) and five (second test) replicated trees ± standard error. For each column, different letters mean significant differences among the fungal isolates according to the Tukey’s HSD test at the probability value of 0.05. Numbers within brackets are the re-isolation percentage of each fungal isolate.

## Data Availability

The original contributions presented in this study are included in the article and Supplementary Material. Further inquiries can be directed to the corresponding authors. Sequences of the selected isolates used in the BLASTn analysis have been deposited in NCBI. Their accession numbers, along with those of the isolates included in the phylogenetic analysis, are provided in Tables S3 and S4, respectively.

## Supplementary Materials

The following are available online at https://www.mdpi.com/article/10.3390/jof12020125/s1. Table S1. Primers used for molecular identification of pomegranate fungal species isolated in this study. Table S2. Comparisons performed in the dual culture assays to study the fungal–fungal interaction. Table S3. Accession numbers and nucleotide sequences from pomegranate fungal pathogens deposited in the NCBI database. Table S4. Accession numbers of the nucleotide sequences used for the phylogenetic analysis deposited in the NCBI database. Figure S1. Phylogenetic trees generated by maximum likelihood (ML) analyses using Concatenated ITS–*TEF1-TUB2* sequences of *Diaporthe* spp. (**a**), *Neopestalotipsis* spp. (**b**), ITS–LSU-*TUB2* of *Sporothrix* spp. (**c**), and ITS-LSU-*TEF1* sequences of *Xenoacremonium* spp. (**d**). *Diaporthella corylina* CBS 121124, *Pseudopestalotiopsis indica* CBS 459.78, *Ophiostoma patagonium* CIEFAP431 and *Acremonium acutatum* CBS 682.71 were used as outgroups, respectively. Type strains were indicated with *. Percentage of replicate trees in which the associated taxa clustered together in the bootstrap test (1000 replicates) are shown on the nodes. Figure S2. Examples of wood browning symptoms observed on pomegranate trees six months after artificial inoculation with *Diaphorthe foeniculina* (**a**), *Neopestalotiopsis rosae* (**b**), *Sporothrix stenoceras* (**c**) and *Xenoacremonium* sp. (**d**), compared with the control (**e**) in the first pathogenicity test.
